# Amyand's Hernia: A Systematic Review of Clinical Presentations, Surgical Management, and Outcomes in an Uncommon Picture of a Common Entity

**DOI:** 10.7759/cureus.92416

**Published:** 2025-09-16

**Authors:** Shikha Tiwari, Ramendra Jauhari, Sumit Bhaskar

**Affiliations:** 1 General Surgery, Ganesh Shankar Vidyarthi Memorial (GSVM) Medical College, Kanpur, IND; 2 Medicine, University College of Medical Sciences, Delhi, IND

**Keywords:** amyand, emergency appendectomy, hernia, mesh inguinal hernioplasty, obstructed inguinal hernia

## Abstract

Amyand's hernia, defined as the presence of the appendix within an inguinal hernia sac, is rare but poses diagnostic and operative challenges. Its management remains controversial, particularly regarding appendectomy and mesh use in contaminated settings. We conducted a Preferred Reporting Items for Systematic Reviews and Meta-Analyses (PRISMA)-guided systematic review of English-language case reports and series published between 2000 and March 2025. Databases were searched using standardized terms; inclusion required cases with operative and outcome details. After excluding review studies and studies for which full articles were not available, we identified seven studies encompassing 17 cases of Amyand's hernia over a 20-year period. Data extracted encompassed demographics, clinical presentation, intraoperative findings per Losanoff-Basson classification, operative technique, mesh usage, postoperative outcomes, follow-up, and recurrence. Classification and contamination-guided strategies remain central. Mesh repair appears safe in clean or minimally contaminated settings, whereas gross contamination favors tissue repair or staged reconstruction.

## Introduction and background

Amyand's hernia is classically defined as the presence of the vermiform appendix within an inguinal hernia sac and has been recognized in surgical literature for nearly three centuries [1,2]. Although uncommon overall, its clinical importance stems from highly variable presentations that range from incidental discovery of a normal appendix to inflamed or perforated appendicitis within the sac [2]. Epidemiologically, Amyand's hernia is estimated to occur in a small fraction of inguinal hernia operations (<1% of all inguinal hernias), and appendicitis within the sac is rarer still, with an incidence of 0.07-0.13%; preoperative diagnosis remains difficult because symptoms overlap with incarcerated or strangulated hernia and classic appendicitis [2].

The most widely used decision framework is the Losanoff-Basson classification, which stratifies cases into Type 1 (normal appendix), Type 2 (acute appendicitis confined to the sac), Type 3 (appendicitis with peritonitis/sepsis), and Type 4 (appendicitis with unrelated intra‑abdominal pathology) to align operative steps with contamination risk [3]. Two modern systematic syntheses, one focused on management implications and one aggregating 111 studies with 161 patients, underscore the heterogeneity of surgical strategies and outcomes, especially regarding appendectomy and prosthetic mesh use [4,5]. Older institutional experiences and case series further illustrate that patient factors (such as age and comorbidity), local tissue status, and surgeon preference influence decisions at the point of care [6].

Since 2020, a steady stream of detailed case reports from diverse settings (Asia-Pacific, Middle East, Europe, Africa, and the Americas) has expanded the spectrum of presentations (e.g., sliding, left-sided, and synchronous femoral hernias) and highlighted laparoscopic and open solutions tailored to contamination and anatomy [7-17]. Taken together, the contemporary question is not whether to use mesh universally, but rather when mesh can be used safely after reduction or appendectomy and when a tissue repair or staged approach is prudent [4,5].

The objective of this review is to synthesize case-based evidence (2000-2025), categorize management patterns by classification and contamination status, and summarize outcomes and recurrence to inform pragmatic decision-making in emergency and elective settings.

## Review

Methods

**Figure 1:**
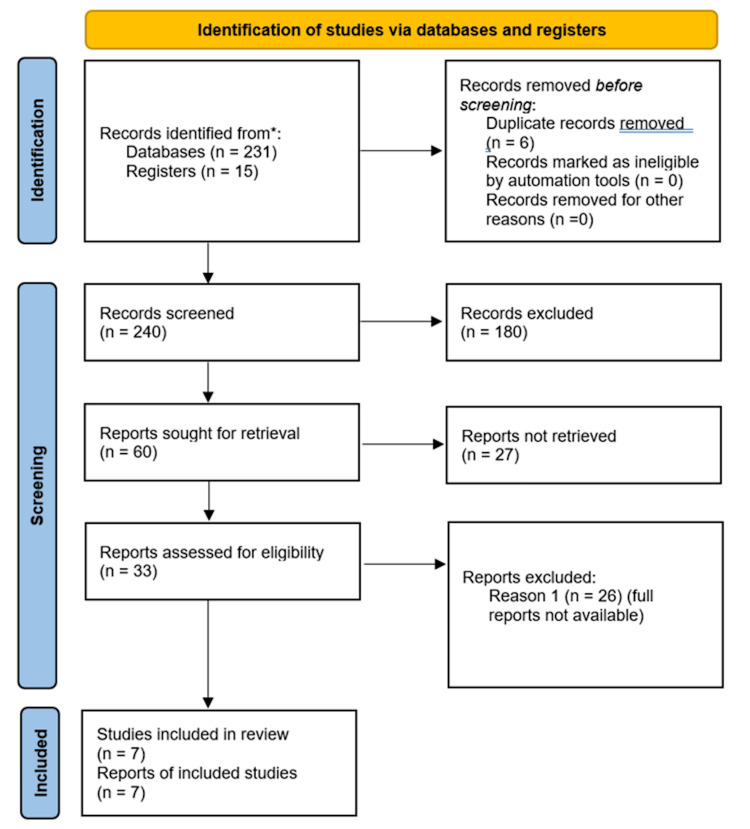
PRISMA flow diagram 2020 PRISMA: Preferred Reporting Items for Systematic Reviews and Meta-Analyses

We followed the Preferred Reporting Items for Systematic Reviews and Meta-Analyses (PRISMA) principles for literature identification, screening, and inclusion, as summarized in Figure 1. We searched PubMed and Embase for English‑language studies from January 2000 through March 2025 using combinations of "Amyand's hernia", "inguinal hernia appendix", and related terms. Studies were included if they (i) reported original clinical data (case reports, case series, retrospective or prospective observational studies, or randomized trials) on patients with Amyand's hernia; (ii) described operative management (open or laparoscopic) with details on appendiceal status (normal, inflamed, or perforated) and hernia repair technique (tissue repair, mesh repair, or staged approach); (iii) provided postoperative outcomes, including morbidity, mortality, wound complications, or recurrence; and (iv) were published in English between January 2000 and March 2025 to capture contemporary surgical practice. Exclusion criteria were (i) reviews, expert opinions, editorials, and conference abstracts without extractable data; (ii) experimental or animal studies; (iii) reports where the appendix was not present in the hernia sac (i.e., not true Amyand's hernia); or (4) studies with insufficient clinical detail to classify cases according to the Losanoff-Basson system.

Database Search Strings

PubMed (searched July 31, 2025) - ("Amyand’s hernia"[MeSH Terms] OR "Amyand hernia"[Title/Abstract] OR "Appendix in inguinal hernia"[Title/Abstract]) AND (case reports[Publication Type] OR case series[Title/Abstract] OR review[Publication Type]) and Embase (searched July 31, 2025) - ('amyand hernia'/exp OR 'amyand hernia':ti,ab OR 'appendix in inguinal hernia':ti,ab) AND ('case report'/de OR 'case series':ti,ab OR 'review'/de).

Study Selection

Two independent reviewers (Reviewer A and Reviewer B) screened titles and abstracts for relevance. Full-text articles were then assessed against inclusion and exclusion criteria. Disagreements were resolved through discussion; if consensus was not reached, a third reviewer (Reviewer C) adjudicated. Disagreements were resolved through discussion, and inter-reviewer agreement was quantified using Cohen's kappa statistic. Duplicate studies were managed manually.

Data Extraction

Data were extracted independently by two reviewers using a predesigned template. Extracted variables included study characteristics (year, country, design), patient demographics, laterality of hernia, appendix status (normal, inflamed, perforated), operative approach (open vs. laparoscopic), hernia repair technique (mesh vs. tissue repair), postoperative outcomes (complications, mortality, recurrence), and follow-up duration.

Data Synthesis

Given the predominance of case reports and small series, a qualitative (narrative) synthesis was performed. Where multiple studies reported comparable outcomes, frequencies and proportions were tabulated to identify patterns in management and outcomes by Losanoff-Basson classification and contamination status. Missing data were managed using list-wise deletion (complete-case analysis). Quantitative meta-analysis was not performed due to heterogeneity of study designs and outcome reporting.

Results

After excluding review studies and studies for which full articles were not available, we identified 17 cases of Amyand's hernia in 20 years. The methodological quality of the included reports was assessed using the JBI Critical Appraisal Checklist for Case Reports/Case Series. Overall, reporting quality was variable: while most provided clear patient demographics and clinical details, many lacked standardized follow-up and outcome reporting, limiting comparability and generalizability. The key reported cases and their clinical characteristics are summarized in Table 1.

**Table 1:** Comprehensive data of all the cases as reported in the previous studies # - No details about whether an appendectomy was performed or not were provided in the study NR* - Not reported in the study References: Cases 1-4, 6, and 7 [18]; case 5 [19]; case 8 [20]; case 9 [21]; cases 10-13 [22]; cases 14-16 [23]; case 17 [24]

Case	Age (Years)	Gender	Clinical Presentation	Intraoperative Appendix Condition	Losanoff-Basson Type	Mesh Used	Post-op Outcome	Follow-Up Duration	Recurrence
1^#^	77	Male	Obstructed right inguinal hernia with right lower quadrant pain	Perforated appendicitis	Type 2	No (Bassini tissue repair)	No complications reported	>1 year	None reported
2^#^	56	Male	Obstructed right inguinal hernia with right lower quadrant pain	Normal appendix	Type 1	Yes (Lichtenstein repair)	No complications reported	>1 year	None reported
3^#^	30	Male	Right lower quadrant pain	Inflamed (non-perforated)	Type 2	Yes (Laparoscopic repair)	No complications reported	>1 year	None reported
4^#^	64	Male	Obstructed right inguinal hernia with pain	Perforated appendicitis	Type 2	Yes (Lichtenstein; polypropylene)	No complications reported	>1 year	None reported
5	61	Male	Enlarging right inguinal hernia x 6 years	Normal appendix	Type 1	Yes (Lichtenstein)	No complications reported	>1 year	None reported
6^#^	77	Male	Obstructed right inguinal hernia with pain	Perforated appendicitis	Type 2	No (laparoscopic appendectomy only)	No complications reported	>1 year	None reported
7^#^	71	Male	Obstructed right inguinal hernia with pain	Normal appendix	Type 1	Yes (Lichtenstein)	Incision infection	>1 year	None reported
8	84	Female	One week of right lower quadrant tenderness, skin changes, and fevers	Perforated appendix with abdominal wall abscess	Type 3	No (only interval appendectomy done)	No complications reported	>1 year	None reported
9	59	Male	Acute abdominal pain, right groin mass	Inflamed (non-perforated)	Type 2	Yes (Lichtenstein repair with appendectomy)	No complications reported	>1 year	None reported
10	2 months	Male	Irreducible right inguinal swelling, with excessive crying x 2 days, with cleft lip and ASD*	Inflamed (Suppurative)	Type 2	No (Appendectomy with herniotomy)	No complications reported	NR*	None reported
11	4	Male	Recurrent right inguinal swelling with irreducibility and mild discomfort	Inflamed appendix	Type 2	No (Appendectomy with herniotomy)	No complications reported	NR*	None reported
12	4 months	Male	Huge, reducible, right inguinal hernia	Normal appendix	Type 1	No (Appendectomy with herniotomy)	No complications reported	NR*	None reported
13	5 months	Male	Bilateral inguinal hernia, cord thickening on the right	Normal appendix	Type 1	No (Appendectomy with herniotomy)	No complications reported	NR*	None reported
14	58	Male	Swelling in the right groin for one month, with pain in the swelling and irreducibility for two days	Inflamed appendix	Type 2	No (Appendectomy with herniorrhaphy)	No complications reported	1 year	None reported
15	42	Male	Swelling in the right groin for six months, with pain in the swelling and irreducibility for the last five days	Perforated appendix	Type 2	No (Appendectomy with herniorrhaphy)	No complications reported	1 year	None reported
16	68	Male	Swelling in the right groin for two months with self-limiting episodes of pain and irreducibility	Normal appendix	Type 1	Yes (Lichtenstein repair without appendectomy)	No complications reported	6 months	None reported
17	35	Male	Right inguinoscrotal swelling for one month with a two-day history of epigastric pain and vomiting	Inflamed appendix	Type 2	No (Bassini's tissue repair with appendectomy)	No complications reported	NR*	None reported

Patients were predominantly male, spanning a wide age range, and most presented with irreducible or painful groin swelling; imaging rarely yielded a definitive preoperative diagnosis, consistent with historical and modern reviews. Intraoperative findings were mapped to the Losanoff-Basson classification, which remained a practical guide to align appendectomy, reduction, and hernia repair strategy with contamination grade. Across reports, surgeons favored mesh hernioplasty when contamination was absent or minimal, including some Type 2 presentations after appendectomy, whereas tissue repairs or staged hernioplasty were preferred in gross contamination or sepsis.

A total of 17 patients with Amyand's hernia were included in this study. The age of the patients ranged from two months to 84 years, with the majority being adults; only four cases occurred in the pediatric age group (<5 years). The cohort was overwhelmingly male-predominant (16 males, one female).

Clinical Presentation

Most patients presented with an irreducible or obstructed right inguinal hernia associated with pain. In pediatric patients, irritability and swelling were the main presenting complaints, whereas adults often reported groin pain, irreducibility, or features suggestive of intestinal obstruction. One elderly female presented with fever, abdominal wall erythema, and a localized abscess.

Intraoperative Findings and Classification

According to the Losanoff-Basson classification, seven patients (41%) had a Type 1 hernia with a normal appendix, while 10 patients (59%) had a Type 2 or 3 hernia, in which the appendix was inflamed or perforated. Specifically, seven patients had an inflamed non-perforated appendix, and five patients had a perforated appendix. One case was associated with an abdominal wall abscess (Type 3).

Surgical Management

The surgical approach varied depending on intraoperative findings and patient factors. Mesh repair (Lichtenstein or laparoscopic technique) was performed in seven patients (41%), mostly in cases with a normal or non-perforated appendix. Tissue repairs (Bassini or herniorrhaphy) and herniorrhaphy with appendectomy were employed in the remaining patients, particularly when appendiceal inflammation or perforation was present. In children, herniorrhaphy with appendectomy or herniotomy was the preferred approach, and mesh was not used.

Postoperative Outcomes

The postoperative course was largely uneventful. Only one patient (6%) developed a superficial surgical site infection following mesh repair, which was managed conservatively. No other early complications, such as seromas, hematomas, or recurrences, were observed.

Follow-Up

The follow-up duration ranged from six months to over one year in adults, while pediatric cases were typically followed for shorter periods. Importantly, no recurrences were reported in any patient during the available follow-up period.

Discussion

Amyand's hernia most often involves a right inguinal hernia sac, reflecting normal appendiceal anatomy; left‑sided cases are exceptional and usually imply underlying anomalies such as situs inversus, intestinal malrotation, or a very mobile cecum [2,16,17].

Preoperative identification is uncommon, as symptoms typically overlap with an incarcerated or strangulated inguinal hernia. Imaging modalities, such as ultrasonography and CT, can suggest the presence of the appendix within the sac, but sensitivity is limited, and most diagnoses are made intraoperatively [2,4,5]. This underscores the importance of intraoperative vigilance, especially when atypical findings such as cecal mobility or unexpected sac contents are encountered.

The literature consistently demonstrates heterogeneity in operative management, reflecting differences in patient age, comorbidities, degree of contamination, and surgeon judgment. While the role of prosthetic mesh has been a recurring focus, contemporary evidence suggests that outcomes are more broadly determined by the adequacy of source control, field cleanliness, and the tailored application of repair techniques [4,5]. Pediatric cases, in particular, differ from adults: appendectomy is often favored even when the appendix appears normal, and mesh use is generally avoided due to growth considerations and infection risk.

Our review also shows an increasing role for laparoscopic approaches, which provide enhanced visualization of the hernia sac, contralateral pathology, and intra-abdominal contamination. This technique has been applied successfully in both adult and pediatric populations, although surgeon expertise remains a limiting factor [8,11,12].

While case reports of Amyand's hernia with sliding or femoral variants have been documented, involvement of the cecum highlights an extreme degree of sac extension and mobility, further complicating intraoperative decision-making. Recognition of such anatomical variants reinforces the need for individualized operative planning and careful assessment of bowel viability before repair [10,11,15-17].

Outcomes

Aggregate evidence from the 20‑year review, supplemented by recent cases, suggests low recurrence and acceptable morbidity when the repair strategy is matched to contamination grade; most favorable outcomes are reported where the prosthesis is avoided in grossly contaminated fields and used judiciously otherwise [4,5,7-12,25-28].

Limitations

Evidence remains dominated by case reports and small series subject to selection and publication biases, heterogeneous follow‑up, and incomplete reporting of contamination grades and antibiotic strategies, which temper the certainty of pooled inferences [4,5].

Practice Implications

A practical approach is to (i) classify intraoperatively, (ii) perform appendectomy when inflamed or perforated, (iii) use mesh in Type 1 and selected Type 2 when contamination is minimal and hemostasis/irrigation are adequate, and (iv) favor tissue repair or staged reconstruction in Type 3-4. Laparoscopy should be considered where expertise exists [3-5,8,11,12].

Overall, the accumulated evidence indicates that favorable outcomes can be achieved when operative decisions are guided not only by the condition of the appendix but also by the broader context of sac contents, contamination, and patient-specific factors. The present case adds to this body of evidence by illustrating an unusual anatomical variant that expands the known spectrum of Amyand's hernia presentations.

## Conclusions

Amyand's hernia is a rare and often unexpected intraoperative finding, with diagnosis before surgery remaining difficult. Management should be tailored to appendix status, contamination, and patient factors rather than a universal approach to mesh use. Pediatric cases warrant special caution, favoring appendectomy and tissue repair. Individualized, classification-guided decision-making remains key to achieving optimal outcomes.

## References

[1] Logan MT, Nottingham JM (2001). Amyand's hernia: a case report of an incarcerated and perforated appendix within an inguinal hernia and review of the literature. Am Surg.

[2] Yagnik VD (2011). Amyand hernia with appendicitis. Clin Pract.

[3] Losanoff JE, Basson MD (2008). Amyand hernia: a classification to improve management. Hernia.

[4] Papaconstantinou D, Garoufalia Z, Kykalos S (2020). Implications of the presence of the vermiform appendix inside an inguinal hernia (Amyand's hernia): a systematic review of the literature. Hernia.

[5] Manatakis DK, Tasis N, Antonopoulou MI (2021). Revisiting Amyand's hernia: a 20‑year systematic review. World J Surg.

[6] Sharma H, Gupta A, Shekhawat NS, Memon B, Memon MA (2007). Amyand's hernia: a report of 18 consecutive patients over a 15-year period. Hernia.

[7] Gujar S, Pawar N, Gupta G, Kuckreja M (2020). Amyand's hernia: a rare case report. Indian J Case Reports.

[8] Kyang LS, See V, Nguyen H (2020). Irreducible inguinal hernia and acute appendicitis: a case of Amyand's hernia. ANZ J Surg.

[9] O'Connor A, John F, Sabri S (2020). Acute appendicitis located within Amyand's hernia—a complex case with concurrent acute cholecystitis. J Surg Case Rep.

[10] Khalid H, Khan NA, Aziz MA (2021). Amyand's hernia a case report. Int J Surg Case Rep.

[11] Elgazar A, Awad AK, Mandal D, Faddah RM, Elder Z, Elseidy SA (2021). Sliding Amyand's hernia: a case report and review of literature. J Surg Case Rep.

[12] Probert S, Ballanamada Appaiah NN, Alam AS, Menon NJ (2022). Amyand's hernia with concurrent appendicitis: a case report and review of the literature. Cureus.

[13] Olamaeian F, Saberi Pirouz M, Sheibani F, Tayebi A (2022). Amyand's hernia: non incarcerated, inflamed appendix in inguinal sac case report. J Surg Case Rep.

[14] Hatampour K, Zamani A, Asil RS, Ebrahimian M (2023). Amyand's hernia in an elective inguinal hernia repair: a case report. Int J Surg Case Rep.

[15] Radboy M, Kalantari ME, Einafshar N, Zandbaf T, Bagherzadeh AA, Shari'at Moghani M (2023). Amyand hernia as a rare cause of abdominal pain: a case report and literature review. Clin Case Rep.

[16] Corvatta FA, Palacios Huatuco RM, Bertone S, Viñas JF (2023). Incarcerated left-sided Amyand's hernia and synchronous ipsilateral femoral hernia: first case report. Surg Case Rep.

[17] Alyahyawi K (2023). Left‑sided Amyand's hernia: a rare variant of inguinal hernia. Cureus.

[18] Gao Y, Zhang T, Zhang M, Hu Z, Li Q, Zhang X (2021). Amyand's hernia: a 10-year experience with 6 cases. BMC Surg.

[19] Green J, Gutwein LG (2013). Amyand's hernia: a rare inguinal hernia. J Surg Case Rep.

[20] Torres Perez-Iglesias C, Kim TD, Marinov M (2023). Amyand's hernia and complicated appendicitis presenting as an abdominal wall abscess. ACS Case Rev.

[21] Youssef Shaban, Adel Elkbuli, Mark McKenney (2018). Amyand’s hernia: a case report and review of the literature. Int J Surg Case Rep.

[22] Shazia Jalil, Muhammad Azhar, Izzat Malkani (2023). Amyand's hernia in children. J Pediatr Surg Case Rep.

[23] Desai G, Suhani Suhani, Pande P, Thomas S (2017). Amyand's hernia: our experience and review of literature. Arq Bras Cir Dig.

[24] da Fonseca-Neto OC, Lucena RC, Lacerda CM (2014). Amyand's hernia: inguinal hernia with acute appendicitis. Arq Bras Cir Dig.

[25] Tawashi K, Khalouf E, Wzet MD, Bittar O (2024). Intraoperative diagnosis of Amyand's hernia, a case report. Int J Surg Case Rep.

[26] Upadrasta VA, Koul A, Chauhan VS (2025). Uncomplicated Amyand's hernia in a setting of abdominal wall insufficiency: a case report. J Med Case Rep.

[27] Khandelwal S, Kaur A, Singh S, Ghosh A (2024). Amyand's hernia: a case of right inguinoscrotal hernia with appendiceal content. Ann Med Surg (Lond).

[28] García Garza JA, Zapata Chavira H (2025). Amyand's hernia with appendicitis in a 64-year-old male: a case report. J Surg Case Rep.

